# Transcriptome dynamics of rooting zone and aboveground parts of cuttings during adventitious root formation in *Cryptomeria japonica* D. Don

**DOI:** 10.1186/s12870-018-1401-7

**Published:** 2018-09-19

**Authors:** Yuki Fukuda, Tomonori Hirao, Kentaro Mishima, Mineko Ohira, Yuichiro Hiraoka, Makoto Takahashi, Atsushi Watanabe

**Affiliations:** 1 Forest Tree Breeding Center, Forestry and Forest Products Research Institute, Forest Research and Management Organization, 3809-1 Ishi, Juo, Hitachi, Ibaraki, 319-1301 Japan; 2 Graduate School of Bioresource and Bioenvironmental Sciences, Kyushu University, 744 Motooka, Nishi-ku, Fukuoka, 819-0395 Japan; 3 Forest Bio-research Center, Forestry and Forest Products Research Institute, Forest Research and Management Organization, 3809-1 Ishi, Juo, Hitachi, Ibaraki, 319-1301 Japan; 4 Faculty of Agriculture, Kyushu University, 744 Motooka, Nishi-ku, Fukuoka, 819-0395 Japan

**Keywords:** *Cryptomeria japonica*, Adventitious root formation, Transcriptome, Conifer, Needles, Microarray

## Abstract

**Background:**

Adventitious root formation is an essential physiological process for successful propagation of cuttings in various plant species. Because coniferous species are highly heterozygous, propagation of cuttings is of great practical use in breeding. Although various factors influence adventitious root formation, little is known of the associated regulatory mechanisms. Whereas adventitious roots generally form from the base of cuttings, this process is accompanied by physiological changes in leaves, which supply assimilates and metabolites. Herein, we present microarray analyses of transcriptome dynamics during adventitious root formation in whole cuttings in the coniferous species, *Cryptomeria japonica*.

**Results:**

Temporal patterns of gene expression were determined in the base, the middle, and needles of cuttings at eight time points during adventitious root formation. Global gene expression at the base had diverged from that in the middle by 3-h post-insertion, and changed little in the subsequent 3-days post-insertion, and global gene expression in needles altered characteristically at 3- and 6-weeks post-insertion. In Gene Ontology enrichment analysis of major gene clusters based on hierarchical clustering, the expression profiles of genes related to carbohydrates, plant hormones, and other categories indicated multiple biological changes that were involved in adventitious root formation.

**Conclusions:**

The present comprehensive transcriptome analyses indicate major transcriptional turning and contribute to the understanding of the biological processes and molecular factors that influence adventitious root formation in *C. japonica*.

**Electronic supplementary material:**

The online version of this article (10.1186/s12870-018-1401-7) contains supplementary material, which is available to authorized users.

## Background

Roots that develop post-embryonically from differentiated cells such as shoots, stems, and leaves, are defined as adventitious roots [1]. Adventitious root formation is an essential physiological process for the successful propagation of cuttings in various horticultural and forest tree species. This vegetative propagation method maintains the genotype of the donor plant, and hence the superior characteristics of targeted clones. Because the resulting uniformity among ramets provides economic benefits for horticultural and forestry industries, numerous investigations have been performed to identify the mechanisms of adventitious root formation. In particular, biochemical studies of horticultural plants have revealed multiple factors that influence adventitious root formation. The plant hormone auxin plays a key role in the onset of adventitious root formation [2–6], and carbohydrates are a clear energy requirement of this process [7–10] (reviewed in [11–14]). Numerous molecular biological studies support biochemical findings in horticultural plants [15–17], reflecting the promise of gene expression profiling for dissecting underlying biological processes in plant physiology and development [18]. Accordingly, previous comprehensive transcriptome analyses have elucidated molecular mechanisms of adventitious root formation [19, 20].

Adventitious roots form under favorable conditions, and vegetative propagation is of great practicable to breed coniferous species, because they are generally highly heterogeneous [21] and have low probabilities of gene fixation. Accordingly, more than 65 million rooted cuttings of coniferous species are produced every year globally, and at least half of these are produced in Japan [22]. Due to its rapid growth, bole straightness and ease of wood processing, *Cryptomeria japonica* is among the most important forestry species in Japan, and has been continuously propagated from cuttings since around the year 1400 [23]. Although *C. japonica* is easily propagated by cuttings, rooting abilities differ considerably with genotype [24], donor tree age [25], and various other factors, and can be enhanced by such treatments with auxin [26] and bottom heating [27]. However, most studies of adventitious root formation in *C. japonica* were conducted using simple binary evaluations of rooting phenotype. Although anatomical changes during adventitious root formation in *C. japonica* have been reported [28], associated molecular events such as changes in gene expression remain poorly understood. In coniferous species, a limited number of molecular biological studies have been conducted and revealed genes that are related to cell replication, cell wall metabolism, auxin metabolism, stress responses, primary carbohydrate metabolism, and photosynthesis were differentially expressed during adventitious root formation following excision from the donor plant [29, 30].

Physiological changes in leaves, which provide assimilates and metabolites, are closely related to adventitious root formation. In particular, auxin has been shown to stimulate adventitious root formation, and is mainly synthesized in leaves and redistributed by a polar auxin transporter [31], and photosynthetic carbohydrate production and transpiration are related to elongation of roots on cuttings [32]. Moreover, studies of *C. japonica* suggested that the conditions around needles of cuttings influence the efficiency of adventitious root formation [33–35]. Adventitious roots generally formed at the base and not in the middle of cuttings, whereas these parts are regarded as equivalent organs before excision. Hence, comparisons of biological changes at the base and the middle of cuttings will contribute to the mechanistic understanding of adventitious root formation.

In this study, we performed transcriptome analyses of cuttings during adventitious root formation with the aim of ﻿providing insight into the molecular mechanisms of this process in *C. japonica*. Three important experimental design elements enhanced the clarity and relevance of our findings as follows: (1) samples were taken from three parts (the base, the middle and needles) of cuttings; (2) samples were collected at eight time points between excision from the donor tree and appearance of adventitious roots; (3) microarray analyses were used to identify global gene expression profiles.

## Methods

### Plant materials and sample collection

**Fig. 1:**
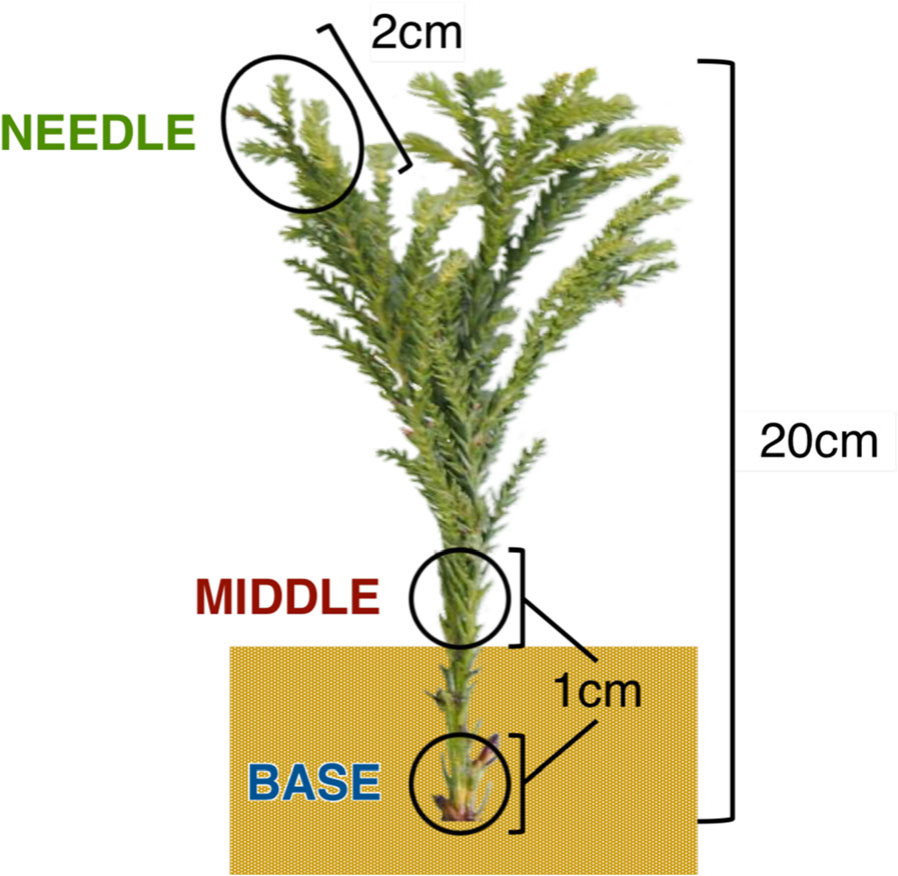
Three parts of *C. japonica* cutting collected for microarray analyses

All samples were taken from individuals of the *C. japonica* plus-tree clone Tsukuba 1. Stem cuttings of about 20 cm in length were taken from 18-year-old donor trees between 9:00 and 10:00 on May 25, 2016, in a field located at the Forest Tree Breeding Center of the Forestry and Forest Products Research Institute (FTBC, Hitachi, Ibaraki, Japan). They were inserted into Kanuma soil, which is a popular substrate for adventitious rooting of *C. japonica*, and were cultivated in a FTBC greenhouse with automatic irrigation and no environmental controls. Appearance of bases of cuttings was observed once weekly to record the timing of rooting. Prior to RNA extraction, a 1-cm length of the base of cuttings (base, underground), a 1-cm length of the middle of cuttings (middle, aboveground) and a 2-cm length of shoots including needles (needles; Fig. 1) were collected at 0 -h post-excision (hpe), 0 -h post-insertion (hpi; about 2 hpe), 3 hpi, 1 -day post-insertion (dpi), 3 dpi, 1 -week post-insertion (wpi), 3 wpi, and 6 wpi. Samples were collected from three cuttings as biological replicates at each time point, leading to a total of 72 samples. Samples were immediately frozen in liquid nitrogen and were stored at − 80 °C until RNA extraction.

### Microarray design

The microarray was designed using e-array (Agilent Technologies, Santa Clara, CA, USA) at the default parameters of the Base Composition Methodology. Isotig sequences for microarray design were collected from various organs (wood, tree-top, shoot, male flower and seedling including root) in several developmental stages and seasons using the Roche GS-FLX system described by Mishima et al. [36]. Probes with high similarity (homology × coverage > 0.95) were excluded and the SurePrint G3 Gene Expression Custom 8 × 60 K Array (Agilent Technologies) comprised three probe sets corresponding to 19,304 sequences. Gene annotations representing the top-scoring BLASTX hits for each sequence were used to predict protein products as a query against the TAIR Arabidopsis protein database TAIR10-pep-20101214 with a threshold e-value of 1e − 5 using the CLC Genomic Workbench version 4.1.1 software package (CLC bio, Aarhus, Denmark).

### RNA extraction and microarray experiments

Total RNA was extracted from samples using an RNeasy Plant Mini kit (Qiagen, Hilden, Germany) with DNase digestion on-column using an RNase-Free DNase Set (Qiagen). Three rooted replicates of the bases of cuttings were used at 6 wpi with a few centimeters of roots. RNA concentrations were measured using a NanoDrop 2000 spectrophotometer (Thermo Fisher Scientific, Waltham, MA, USA) and RNA integrity was assessed using an Agilent 2100 Bioanalyzer (Agilent Technologies) with an Agilent RNA 6000 Nano Kit (Agilent Technologies). Cyanine-3 (Cy3) labeled cRNA was then synthesized from 200 ng of total RNA from each sample with a Low Input Quick Amp Labeling Kit (Agilent Technologies), and 600 ng of the Cy3-labeled cRNA was fragmented at 60 °C for 30 min and was hybridized to 8 × 60 K microarray for 17 h at 65 °C in a rotating microarray hybridization oven (Agilent Technologies) with a Gene Expression Hybridization Kit (Agilent Technologies). Following hybridization, microarray slides were washed with a Gene Expression Wash Buffer Kit (Agilent Technologies) and scanned using a SureScan Microarray Scanner version 9.1 (Agilent Technologies). Signal intensities of the spots on the resulting TIFF images were quantified using Feature Extraction software version 11.5 (Agilent Technologies) and were used to calculate gene expression levels. Details of the microarray design and the ensuing data have been submitted to the NCBI GEO database [GSE102874].

### Processing of microarray data

**Fig. 2:**
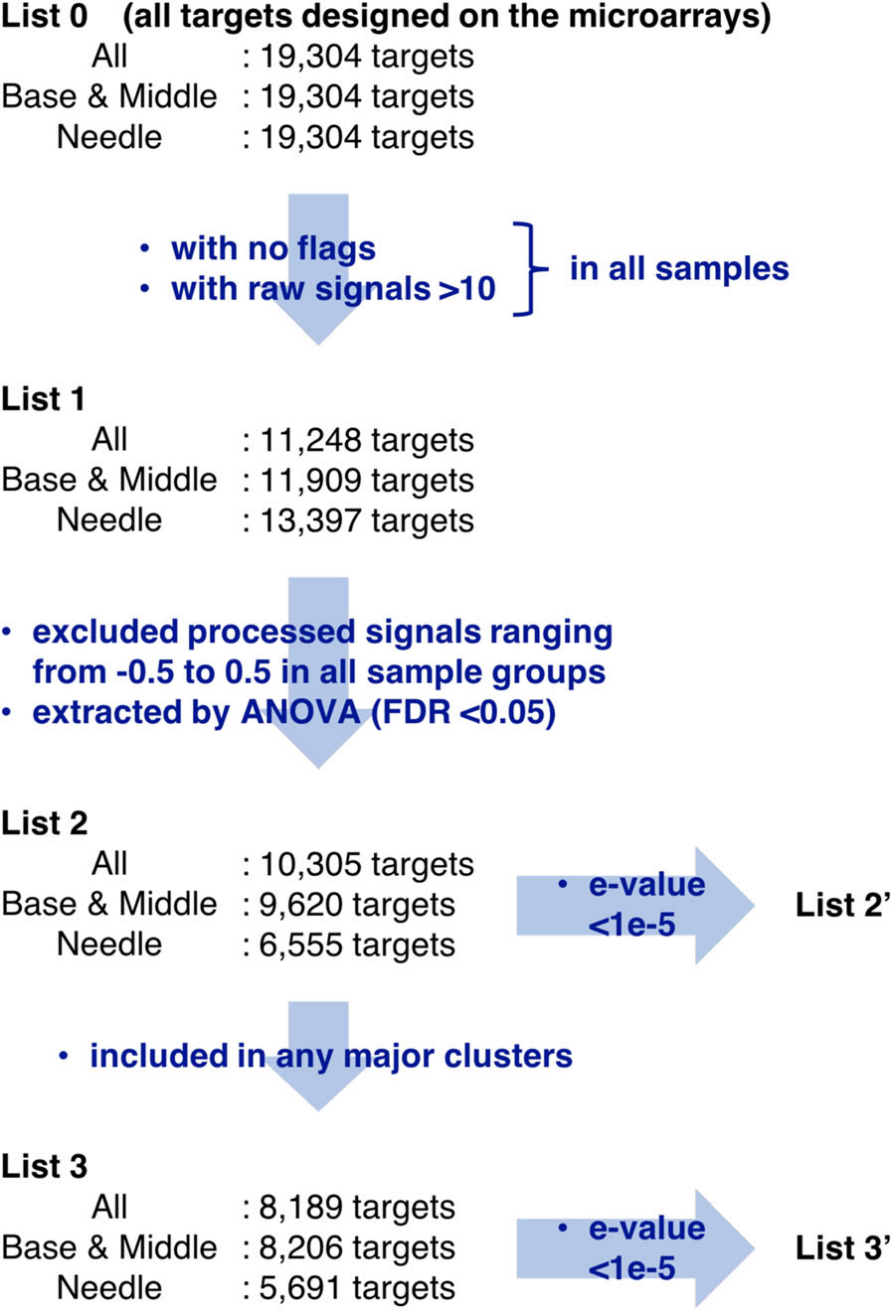
Data processing

In order to investigate changes in gene expression in whole cuttings and individual parts, we constructed and processed three separate datasets for all parts, the base and the middle, and needles, with consideration of the influence of differences in global gene expression on normalization (Fig. 2). The present 19,304 targets (list 0) were spotted in three replicates on the microarray and mean signal intensities were considered quantitative of gene expression levels in each sample. The expression levels of list 0 were normalized using log_2_-transformation and global normalization to the 75th percentile using the Subio platform version 1.21 (Subio Inc., Kagoshima, Japan). To improve data quality, targets (list 1) with unfavorable flags (gIsFeatNonUnifOLs, gIsBGNonUnifOLs, and gIsWellAboveBGs) in any replicated spots were identified and excluded using Feature Extraction software, and analyses were only performed using targets with mean raw expression levels of greater than 10 in all samples. Mean expression levels for each sample group were considered representing gene expression levels and targets (list 2) with expression levels that varied for at least one sample group were extracted by one-way analysis of variance (Benjamini–Hochberg false discovery rate < 0.05) using targets whose processed expression levels were less than − 0.5 or greater than 0.5 in at least one group. Principal component analysis (PCA) and hierarchical clustering were then conducted using the unweighted pair group method with arithmetic mean and Pearson’s correlations, and a general overview of list 2 was generated. Based on hierarchical clustering, 13, 12, and 8 major clusters were identified from the three datasets, respectively, and targets (list 3′) with hits for unique *Arabidopsis thaliana* genes with an e-value <1e − 5 after BLASTX searches were considered significantly homologous and were selected from each cluster to estimate gene annotations in *C. japonica*. These targets were then identified with gene names and were categorized into GOTERM_BP_DIRECT using the Database for Annotation, Visualization and Integrated Discovery (DAVID 6.8, https://david.ncifcrf.gov) [37]. For analysis of Gene Ontology (GO) term enrichment, the EASE scores, which are modified Fisher exact *p*-values [37], were used to define gene enrichments that were associated with annotation terms using the targets (list 1′) with lower e-values (<1e − 5) in list 1 as the population background.

### Quantitative RT-PCR

Microarray analyses were independently verified using qRT-PCR (Additional file 1: Figure S1, Additional file 2: Figure S1 and Additional file 3: Figure S3) with primers that were designed using Primer Express software v3.0 (Applied Biosystems, Foster City, CA, USA; Additional file 4: Table S1). Amplification efficiencies of all primer pairs were optimized using genomic DNA from the clone tree that was used for microarray experiments on the StepOnePlus Real-Time PCR System (Applied Biosystems) using SYBR Green real-time qRT-PCR. Total RNA (160 ng) was then reverse-transcribed using a High-Capacity RNA-to-cDNA Kit (Applied Biosystems) according to the manufacturer’s instructions. PCR mixtures were prepared according to the manufacturer’s instructions and contained forward and reverse primers at 250 nM and 5 μL of 43-fold diluted cDNA in a final volume of 20 μL. All reactions were denatured at 95 °C for 10 min, followed by 40 cycles of 95 °C for 15 s and 60 °C for 1 min. Melting curve analyses were then performed from 60 °C to 95 °C and data were captured every 0.3 °C to ensure amplification of single products. Relative quantifications and comparisons, were performed using the delta-delta-Ct method with *Ubiquitin* expression as the internal control [38, 39].

## Results and discussion

### Overall assessment of gene expression changes during adventitious root formation in coniferous cuttings

**Fig. 3:**
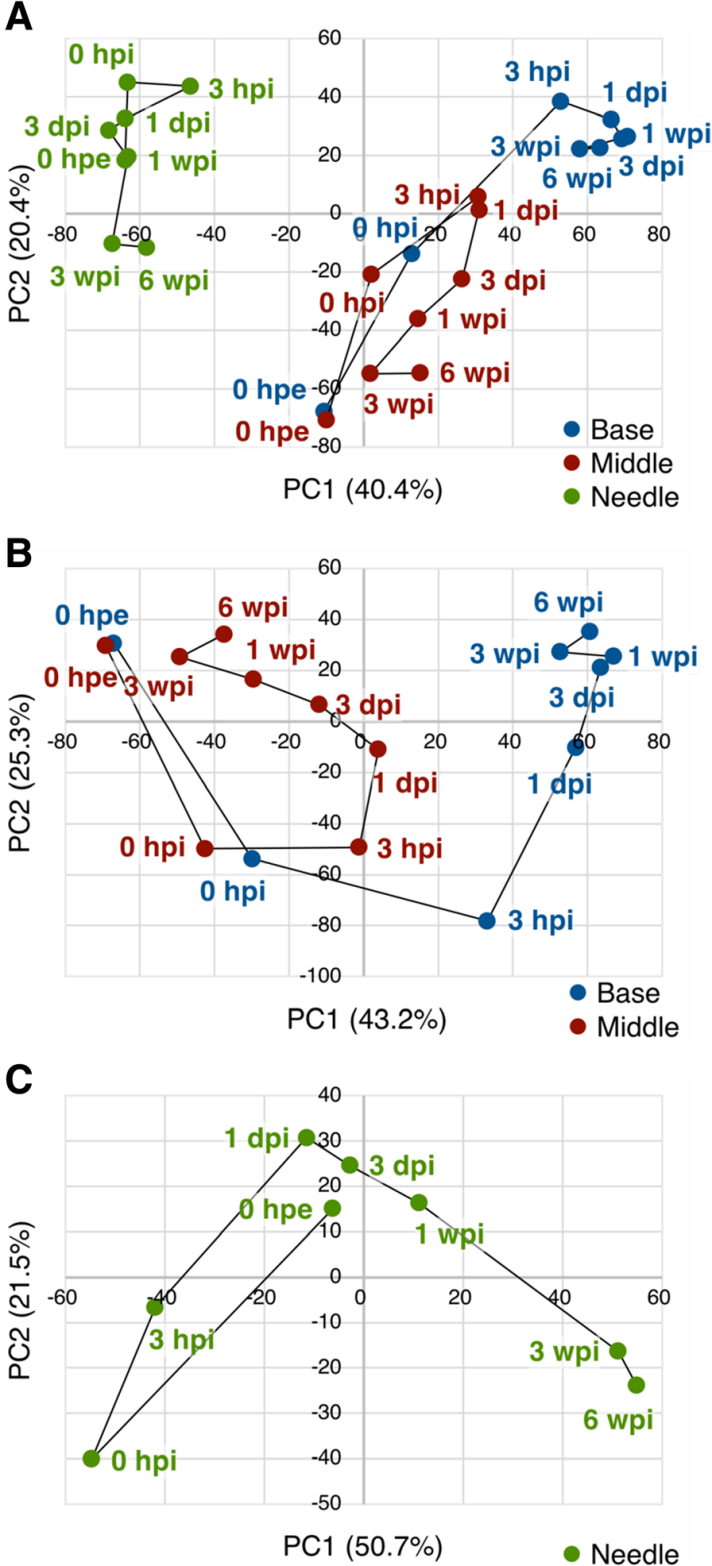
Principal component analysis of microarray data. The plot illustrates the principal components of (**a**) all 24 sample groups for 10,327 genes in “all”, (**b**) all 16 sample groups for 9,633 genes in “base and middle” and (**c**) all 8 sample groups for 6,555 genes in “needles” that were differentially expressed among time points. Abbreviations for time points are as described in the Methods

**Fig. 4:**
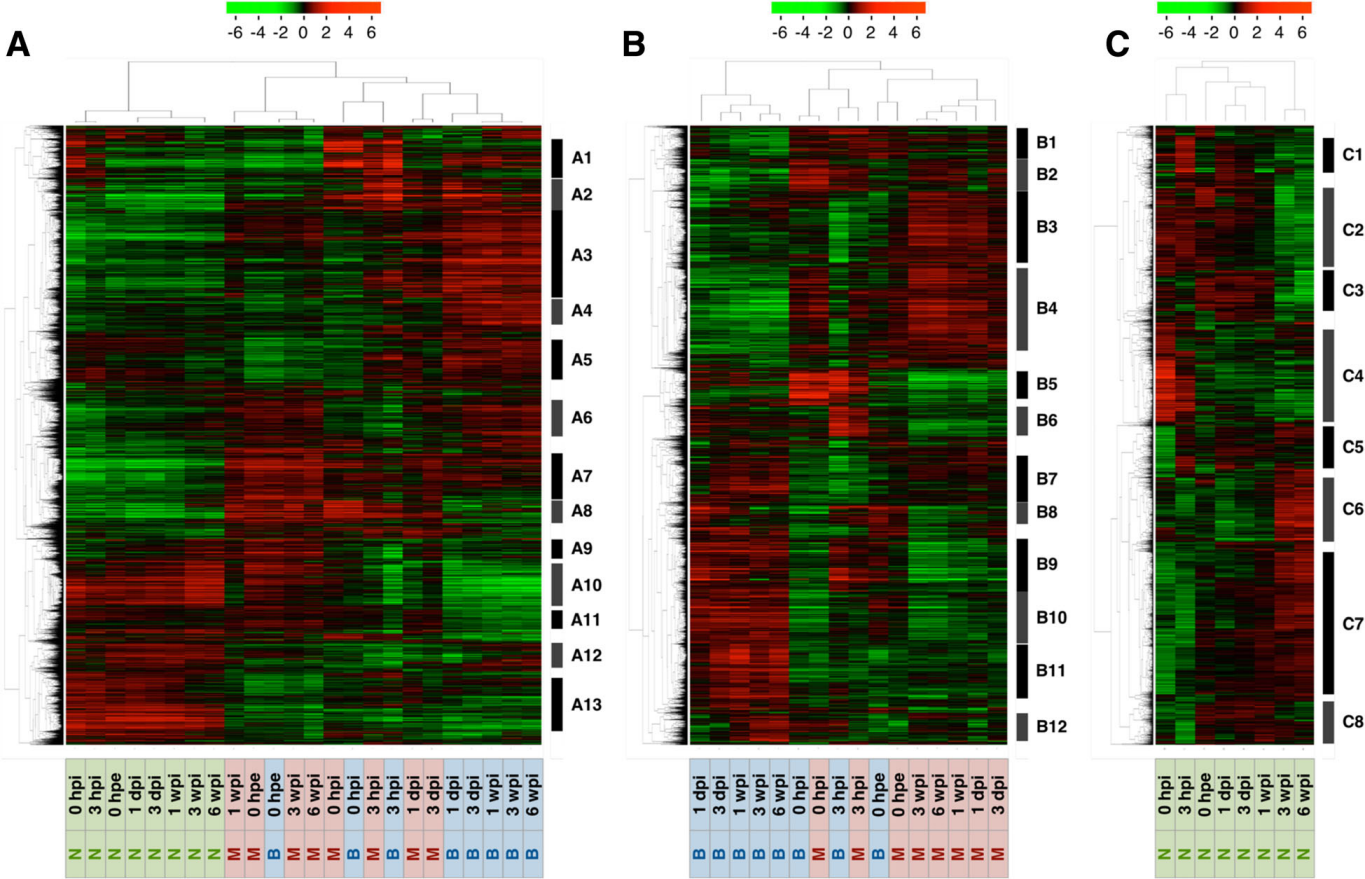
Hierarchical clustering analyses of differentially expressed genes among time points. Heatmaps include (**a**) all 24 sample groups for 10,327 genes in “all”, (**b**) all 16 sample groups for 9,633 genes in “base and middle” and (**c**) all 8 sample groups for 6,555 genes in “needles”, respectively. Labeled bars indicate distinct clusters that were used in further analyses. Color strengths of red and green indicate up- and down-regulation of gene expression level, respectively. Abbreviations for time points are described in the methods; B, base of cuttings; M, middle of cuttings; N, needles

**Fig. 5:**
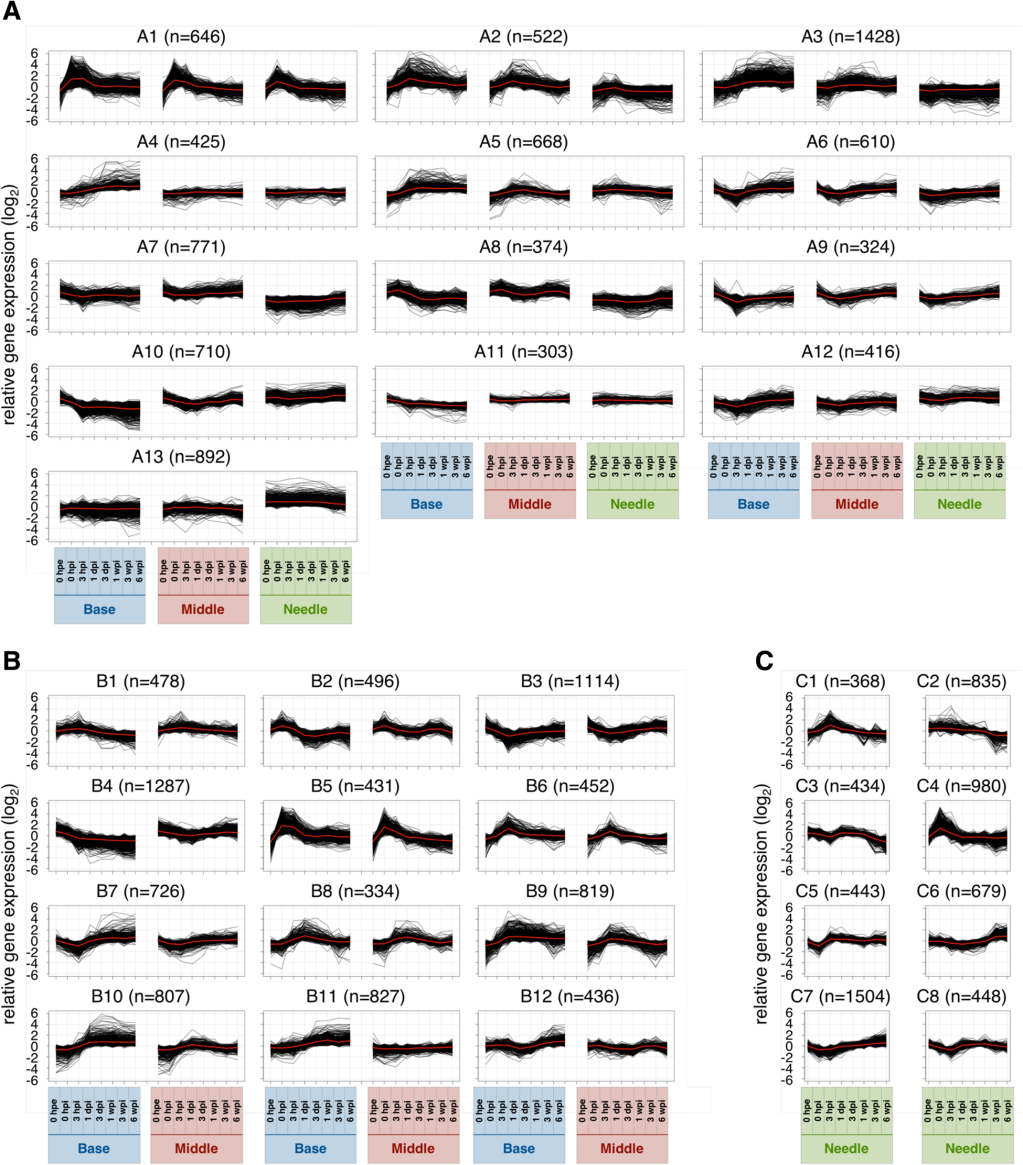
Expression profiles of major clusters. Expression levels of transcripts that varied for at least one sample group were categorized into (**a**) 13 groups in “all”, (**b**) 12 groups in “base and middle” and (**c**) 8 groups in “needles” based on Pearson’s correlations. Red lines indicate the average expression profiles of each cluster. Abbreviations for time points are as described in the Methods

To summarize similarities between samples according to changes in gene expression levels, we performed PCA and hierarchical clustering and characterized gene expression patterns in parts of cuttings during adventitious root formation (Figs. 3a and 4a). Global gene expression levels differed between needles and the other two parts, and were similar at 0 hpe but became more different over time between the base and the middle. Moreover, based on hierarchical clustering, thirteen major clusters were identified (Figs. 4a and 5a and Additional file 5: Table S2A). Annotations of genes in these clusters are listed in Additional file 5 and GO enrichment analysis of each cluster is shown in Additional file 6.

**Fig. 6:**
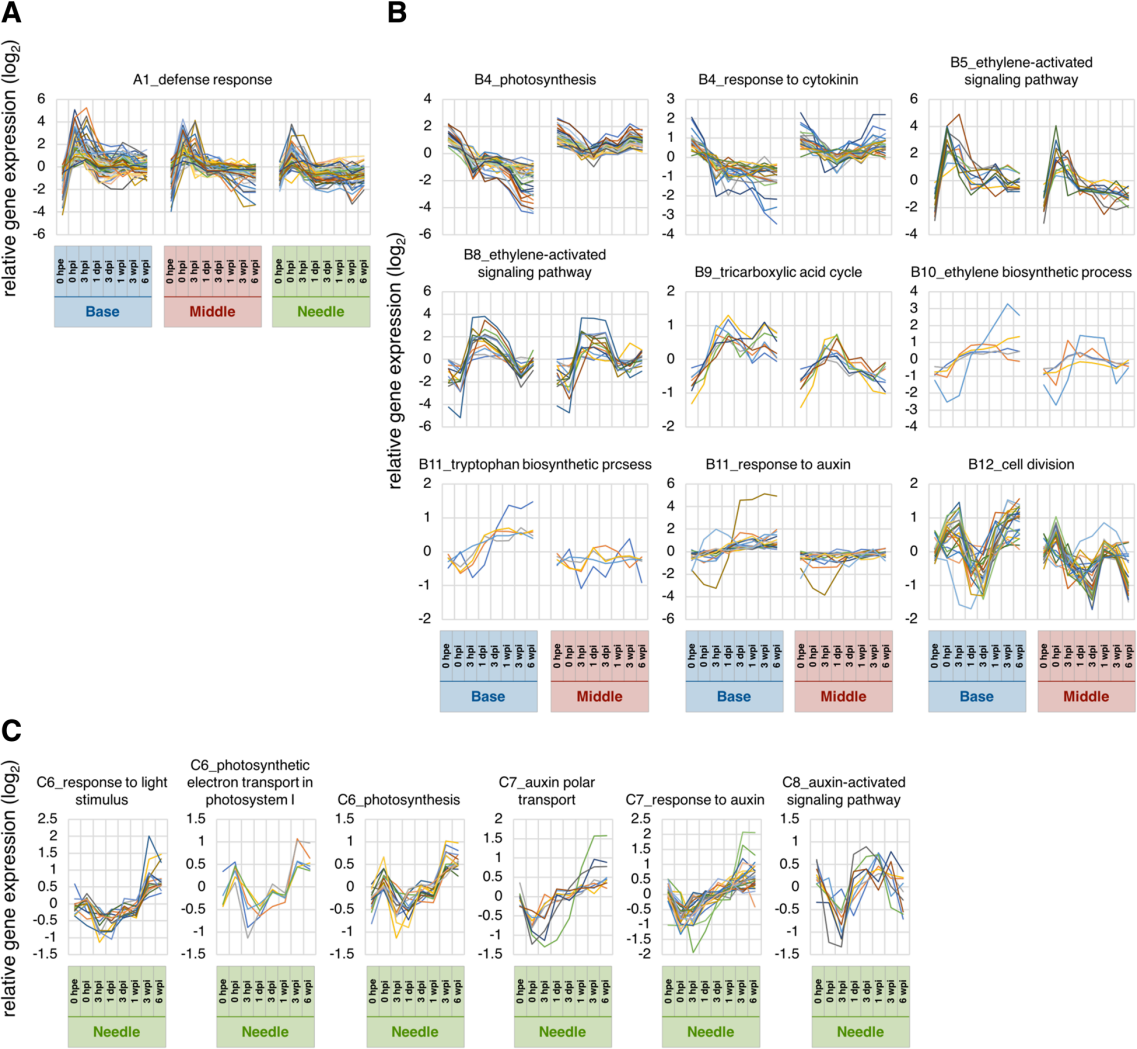
Expression profiles of specific terms in major clusters. Line graphs show expression levels of specific terms in major clusters for (**a**) “all”, (**b**) “base and middle” and (**c**) “needles”. Abbreviations for time points are as described in the Methods

Cluster A1 showed a commonly marked peak at 0 hpi and was the most enriched in genes related to defense response (Fig. 6a and Additional file 6: Table S3A), suggesting that wound signals due to excision are transmitted to the whole plant body and induce immediate transcriptional responses. Clusters A3, A7, and A13 had differing gene expression patterns between needles and the other two parts. However, clusters A7 and A13 maintained the initial difference, whereas cluster A3 increased after excision in the base and the middle but changed little in needles. Hence, clusters A7 and A13 likely reflected original differences in biogenic roles and cluster A3 is relatively important for adventitious root formation. Cluster A3 was enriched in genes related to mRNA processing (GO:0006397), transcription, DNA-templated (GO:0006351), rRNA processing (GO:0006364), embryo sac development (GO:0009553), regulation of transcription, DNA-templated (GO:0006355), negative regulation of transcription, DNA-templated (GO:0045892), RNA splicing (GO:0008380), and RNA secondary structure unwinding (GO:0010501; Additional file 6: Table S3A), suggesting that biological changes are more notable in the base and the middle. Cluster A7 was more highly expressed in the base and the middle than in needles at all time points, and was enriched in genes related to signal transduction (GO:0007165), defense response (GO:0006952), protein retention in Golgi apparatus (GO:0045053), reciprocal meiotic recombination (GO:0007131), and response to auxin (GO:0009733; Additional file 6: Table S3A). Cluster A13 was expressed at lower levels in the base and the middle than in needles at all time points, was enriched in genes related to translation (GO:0006412), fatty acid biosynthetic process (GO:0006633), cell wall organization (GO:0071555), cell redox homeostasis (GO:0045454), ribosome biogenesis (GO:0042254), ATP synthesis coupled proton transport (GO:0015986), glycerol ether metabolic process (GO:0006662), sulfate assimilation (GO:0000103), transport (GO:0006810), and cellular response to oxidative stress (GO:0034599; Additional file 6: Table S3A). Clusters A4 and A10 differed significantly between the base and the middle of the cuttings. Specifically, cluster A4 was gradually up-regulated only at the base, and was enriched in genes related to vegetative to reproductive phase transition of meristem (GO:0010228), leaf senescence (GO:0010150), and ethylene biosynthetic process (GO:0009693; Additional file 6: Table S3A). Cluster A10 was gradually down-regulated only at the base, and was more enriched in genes related to photosynthesis (GO:0015979), oxidation-reduction process (GO:0055114), chlorophyll biosynthetic process (GO:0015995), thylakoid membrane organization (GO:0010027), chloroplast organization (GO:0009658), and protein-chromophore linkage (GO:0018298; Additional file 6: Table S3A). The genes included in these terms are likely responsible for adventitious roots being formed from the base only.

### Gene expression profiles in the base and the middle of cuttings during adventitious root formation

**Fig. 7:**
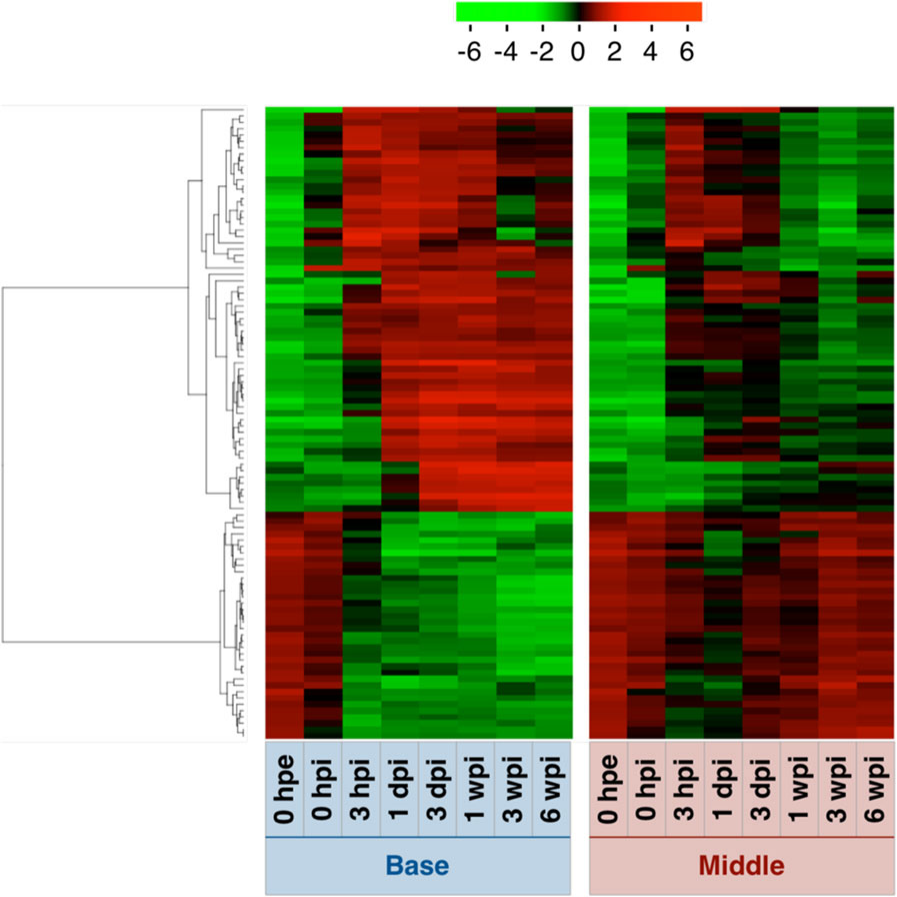
Heatmap of expression of the top 100 genes with the largest PC1. Abbreviations for time points are as described in the Methods

PCA and hierarchical clustering demonstrated remarkable similarities of global gene expression in the base and the middle at 0 hpe (Figs. 3b and 4b). After excision, gene expression levels in both the base and the middle initially showed more dependent on principal component 1 (PC1, 43.2%) and dependence on principal component 2 (PC2, 25.3%) decreased substantially before recovering after 3 hpi; however, the dependence on PC1 continued to increase for the base but decreased for the middle, thus tending to more closely resemble original dependences. A total of 68.5% of transcriptome alterations in these two parts were represented by PC1 and PC2, and PC1 seemed to represent differences between the base and the middle. The top 100 genes with the largest PC1 coefficients and described with their annotations in Additional file 7, and corresponding expression patterns are illustrated in Fig. 7. These genes could be divided into two major expression patterns. One was genes induced after excision that were maintained at the base but not in the middle, and the other was genes repressed after excision that were maintained at the base but not in the middle (Fig. 7). The former was enriched in genes related to oxidation-reduction process (GO:0055114), flavonoid biosynthetic process (GO:0009813), and metabolic process (GO:0008152), and the latter was enriched in genes related to photosynthesis (GO:0015979) and response to light stimulus (GO:0009416; Additional file 8: Table S5). These results indicate that the base and the middle of cuttings were originally equivalent organs and diverged into distinct organs due to physical and physiological stimuli such as excision and insertion. Additionally, twelve major clusters were identified based on hierarchical clustering (Figs. 4b and 5b and Additional file 5: Table S2B). Annotations of genes in these clusters are listed in Additional file 5 and the results of corresponding GO enrichment analyses are presented in Additional file 6. Clusters B4, B11, and B12 showed different expression patterns between the base and the middle. Cluster B4 was enriched in genes related to photosynthesis (GO:0015979) and oxidation-reduction process (GO:0055114), cluster B11 was enriched in genes related to regulation of transcription, DNA-templated (GO:0006355), and cluster B12 was enriched in genes related to microtubule-based movement (GO:0007018), DNA replication (GO:0006260), cell division (GO:0051301), DNA recombination (GO:0006310), mitotic chromosome condensation (GO:0007076), cell cycle (GO:0007049), DNA replication initiation (GO:0006270), mitotic nuclear division (GO:0000278), and regulation of cell cycle (GO:0051726; Additional file 6: Table S3B). Considering that these two parts developed morphological differences, genes related to these terms may have more important roles in adventitious root formation. Moreover, adventitious root formation involves the construction and development of new root primordia from differentiated cells, and thus cell division is indispensable for this process. Previous studies also confirmed altered expression of genes involved in processes such as cell division genes in the rooting zones during adventitious root formation in *P. hybrida* [20] and *P. contorta* [29], and predominantly agree with the present data.

### Temporal changes in gene expression at the base of cuttings during adventitious root formation

Gene expression profiles at the base, which is the rooting zone, were distinguished from those in the middle at 3 hpi, although changed relatively little after 3 dpi (Fig. 3b), suggesting that the transcriptional conditions for adventitious root formation had been established. It is widely recognized that adventitious root formation is histologically categorized into induction, initiation, and extension phases [40]. Satoo [28] reported that the initiation of root primordia formation, which seemed to represent the transition from induction to initiation phases, occurred at 18 dpi and adventitious roots emerged from the cortex at 40 dpi in *C. japonica*. Because adventitious roots emerged at 35 dpi in the present study, histological change of our samples likely occurred around 18 dpi. Thus, our results indicated a large time between the establishment of stable gene expression toward adventitious root formation and the ensuing histological traits. These results also indicate that more important gene expression changes occur during the induction phase, and that the major turning point in adventitious root formation occurs long before adventitious root emergence in *C. japonica*.

### Gene expression patterns in needles during adventitious root formation

The present hierarchical clustering analyses generated eight major clusters (Figs. 4c and 5c and Additional file 5: Table S2C), and the included genes are annotated in Additional file 5 and the results of GO enrichment analyses of each cluster are presented in Additional file 6. PCA showed that transcriptome changes in needles occurred in three phases except for 0 hpe (Fig. 3C). Global gene expression in needles altered significantly at 0 and 3 hpi, and then return to the level close to 0 hpe. We assumed that these changes represented responses to excision and corresponding restoration because cluster C4, which peaked at 0 hpi, was most enriched in genes involved in defense responses (GO:0006952; Additional file 6: Table S3C). Global gene expression from 1 dpi to 1 wpi changed little and was similar to those at 0 hpe in needles (Figs. 3c and 4c), whereas actively changed at the base (Figs. 3b and 4b). These results suggest that needles of cuttings retain similar functions to those before excision, even following severe event such as excision from a donor tree that immediately reduces water absorption ability. In contrast, characteristic changes of gene expression at 3 and 6 wpi may represent the biological changes in the plant body. Accordingly, clusters C2, C3 and C6 showed characteristic alterations at 3 and 6 wpi (Figs. 4c and 5c). Among these, cluster C2 was repressed after 3 wpi and was more enriched in genes related to translation (GO:0006412), cell division (GO:0051301), cytoplasmic translation (GO:0002181), ribosome biogenesis (GO:0042254), regulation of cell cycle (GO:0051726), and microtubule-based movement (GO:0007018), whereas cluster B3 was also repressed after 3 wpi was enriched in genes related to lipid catabolic process (GO:0016042), wax biosynthetic process (GO:0010025), oxidation-reduction process (GO:0055114), cuticle development (GO:0042335), DNA replication initiation (GO:0006270), and fatty acid biosynthetic process (GO:0006633; Additional file 6: Table S3C). Cluster C6 was induced after 3 wpi and was enriched in genes related to oxidation-reduction process (GO:0055114), and photosynthesis (GO:0015979; Additional file 6: Table S3B). The observation that the expression of genes involved in cell division, biosynthetic and catabolic processing of secondary metabolites, and photosynthesis changed markedly at 3 and 6 wpi indicates that needles are likely not completely unrelated to adventitious root formation in *C. japonica*.

### Energy metabolism related genes

Carbohydrates are the principal energy source for living organisms and strongly influence adventitious root formation by providing energy for cell growth [7, 8, 10]. .Previous studies have shown that photosynthesis in leaves is related to adventitious root formation [41, 42], although changes in photosynthetic rates during adventitious root formation differ depending on plant species and the season of excision [17, 43, 44]. In *C. japonica*, photosynthetic rates decreased moderately and were restored after rooting in a growth chamber under fixed conditions [45]. In our analyses, genes related to photosynthesis (GO:0015979) photosynthetic electron transport in photosystem I (GO:0009773), and response to light stimulus (GO:0009416) were enriched in cluster C6 (Fig. 6c, Additional file 6: Table S3C), which was induced after 3 wpi in needles. These data indicate no marked changes in photosynthetic activities throughout the induction phase in *C. japonica*. These results also indicate that carbohydrates are not produced specifically for adventitious root formation, and thus the original photosynthetic activities and carbohydrate contents of cuttings are a more important factor in the induction of adventitious root formation, at least in *C. japonica*. However, characteristic induction of these genes after 3 wpi may represent an influence of carbohydrates on the initiation and extension of adventitious roots, warrant further studies of energy metabolism during adventitious root formation.

Genes related to photosynthesis (GO:0015979, Fig. 6b) and some related terms were enriched in cluster C4 (Additional file 6: Table S3C), and showed a marked gradual decrease in expression over time at the base. Although this decrease may have been caused by insertion of the cutting into soil, similar findings have been obtained in cuttings of other species such as *P. contorta* [29], *Vigna radiata* [46] and, *Dianthus caryophyllus* [47], suggesting that the decline in photosynthetic activity is one of the physiological changes associated with adventitious root formation [9]. Moreover, genes related to tricarboxylic acid cycle (GO:0006099, Fig. 6b), were enriched in cluster B9 (Additional file 6: Table S3B), which was up-regulated until 3 hpi and was then maintained at the same level. Respiration pathways, including the tricarboxylic acid cycle, play central roles in carbon metabolism and bioenergetics in aerobic organisms and are essential for growth, maintenance, and carbon balance of all plant cells [48]. Ahkami et al. [9] revealed changes in the activities of key enzymes and metabolites from the tricarboxylic acid cycle and glycolysis during adventitious root formation, and suggested a relation of respiratory function to adventitious root formation in *P. hybrida*. Our results show decreased expression of photosynthetic genes and increased expression of tricarboxylic acid cycle genes at the base but not in the middle, suggesting a functional transition of the base to a sink organ. Hence, these data confirm that adventitious root formation is an energy-requiring process.

### Plant hormonal metabolism related genes

**Fig. 8:**
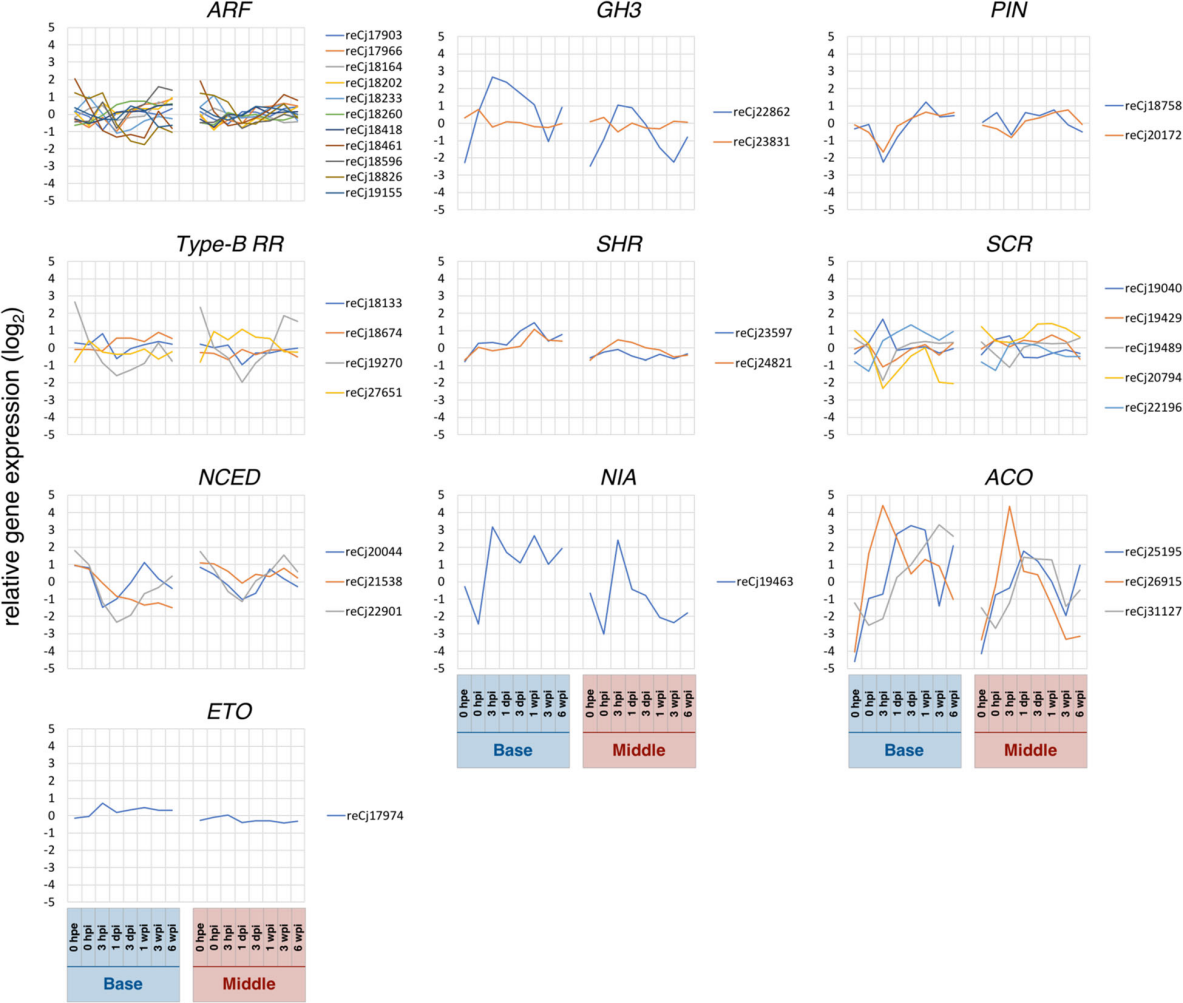
Expression patterns of candidate genes related to adventitious root formation. Abbreviations for time points are as described in the Methods

Herein, we provide transcriptomic evidence of plant hormonal metabolism during adventitious root formation in *C. japonica*. In particular, genes related to tryptophan biosynthetic process (GO:0000162, Fig. 6b) and response to auxin (GO:0009733, Fig. 6b) were enriched in cluster B11, which showed gradual increases in expression over time at the base (Additional file 6: Table S3B). Genes related to auxin polar transport (GO:0009926, Fig. 6c) and response to auxin (GO:0009733, Fig. 6c) were enriched in cluster C7, which showed gradual increases in expression after 1 dpi. In addition, genes related to the auxin-activated signaling pathway (GO:0009734, Fig. 6c) were enriched in cluster C8, which showed transient decreases in expression at 3 hpi (Additional file 6: Table S3C). Cluster C7 also included the *ALDEHYDE OXIDASE* gene, which has been strongly associated with auxin biosynthesis [49]. Numerous studies have shown that auxin is closely related to the onset of adventitious root formation [2–6]. Furthermore, Garrido et al. [31] showed that auxin which is involved in adventitious root formation is mainly synthesized in leaves prior to transport through the polar auxin transport pathway in *D. caryophyllus*. Herein, eleven homologs of *AUXIN RESPONSE FACTORS* (*ARFs*) were included in list 2′ for “base and middle”, and these had diverse gene expression profiles (Fig. 8). These transcription factors regulate the expression of auxin response genes, such as *AUX/IAA*, *SMALL AUXIN UP RNA* (*SAUR*), and *GRETCHEN HAGEN 3* (*GH3*) by binding auxin response elements in the promoter regions of auxin-inducible genes, and thus function as transcriptional activators and repressors [50]. *GH3* genes are reported molecular markers for monitoring changes in auxin concentrations or cellular sensitivity to auxin [51], and Gutierrez et al. [4] showed that these molecules affect adventitious root formation by modulating jasmonic acid homeostasis in *A. thaliana*. Similarly, the auxin efflux carrier encoded by *PIN FORMED* (*PIN*) has known important roles in adventitious root formation [52, 53]. Moreover, one of *GH3* homologs was included in list 2′ for “base and middle”, and showed a highly significant increase in expression after excision, supporting its roles as a candidate gene for adventitious root formation in *C. japonica* (Fig. 8). Transcription from the single homolog of the *PIN* gene which was included in list 2′ for “base and middle” was also significantly induced at 3 hpi and increased expression was observed thereafter (Fig. 8). Ahkami et al. [5] revealed that indole-3-acetic acid concentrations in petunia cuttings increases during the first 2 hpe, transiently decrease until 6 hpe and are then reached a maximal at 24 hpe at the base, and maintained an initial level until 48 hpe and continuously increase thereafter in leaves. Although time scales vary between species, our transcriptome data are comparable with these previous biochemical findings, and indicate that auxin plays positive roles in adventitious root formation.

Cytokinin influences the auxin signaling pathway [54, 55] and inhibits adventitious root formation. Accordingly, Villacorta-Martin et al. [47] showed lower concentrations of the cytokinin *trans*-zeatin in an easy-to-root clone of carnation between 0 and 54 hpi, suggesting that adventitious root formation is related to ratios of auxin and cytokinin. In our hands, genes that are related to response to cytokinin (GO:0009735, Fig. 6b) were enriched in cluster B4 (Additional file 6: Table S3B), and were gradually suppressed over time at the base. In addition, four homologs of *CYTOKININ TYPE-B RESPONSE REGULATOR* (*TYPE-B RR*) were included in list 2′ for “base and middle”. This gene acts as a positive regulator of cytokinin signaling, and thus as a negative regulator of adventitious root formation [56]. We show that one of the four homologs (reCj19270) was greatly repressed until 1 dpi (Fig. 8), suggesting that this homolog is related to adventitious root formation in *C. japonica*. Combined with expression data for genes related to response to auxin and response to cytokinin, our transcriptome analyses suggest that the interplay between auxin and cytokinin is important for adventitious root formation in coniferous trees, as shown previously in herbaceous plants [47, 56].

The plant hormone ethylene has also been related to auxin metabolism during adventitious root formation [57, 58], and is reportedly induced by wounding [59, 60]. In contrast to cytokinin, ethylene plays a positive role in negatively regulating the free auxin accumulation, positively regulating auxin transport in roots, and negatively regulating auxin transport in shoots [61]. In this study, expression patterns of genes related to ethylene metabolism were divided into three major clusters; genes related to ethylene-activated signaling pathway (GO:0009873, Fig. 6b) were enriched in cluster B8, those related to ethylene biosynthetic process (GO:0009693, Fig. 6b) were enriched in cluster B10, and those related to ethylene-activated signaling pathway (GO:0009873, Fig. 6b) were enriched in cluster B5 (Additional file 6: Table S3B). The expression levels of these genes were dramatically and characteristically altered at the base during adventitious root formation, and altered in a similar pattern in the middle. In contrast, expression patterns of the ethylene biosynthetic regulatory homologs *ACO* and *ETO* from list 2′ for “base and middle” [62–64] differed between the base and the middle. Although these gene expression levels at the base suggest relationships with adventitious root formation, further studies are required to reveal their roles and interactions with those in the middle.

### Other candidate genes for adventitious root formation

Functional groups of differentially expressed genes and their related cellular events during adventitious root formation have been reviewed previously [65]. Of these and their families, *SHORT-ROOT* (*SHR*), *SCARECROW* (*SCR*), *TYPE-B RR*, *NINE-CIS-EPOXYCAROTENOID DIOXYGENASE* (*NCED*), *ARF*, *GH3*, *PIN*, *NITRATE REDUCTASE* (*NIA*), *1-AMINOCYCLOPROPANE-1-CARBOXYLATE OXIDASE* (*ACO*), and *ETHYLENE OVERPRODUCER* (*ETO*) were included in the present list 2′ for “base and middle”, and their gene expression patterns and annotations are shown in Fig. 8 and Additional file 9, respectively *SHR* and *SCR* are members of the *GRAS* family and are involved in root stem cell niche specification and asymmetric cell division, and *SHR* activity is necessary for *SCR* expression [66]. Herein, two *SHR* homologs and five *SCR* homologs were included in list 2′ for “base and middle”. Expression of the *SHR* homologs reCj23597 showed significant differences except at 0 hpe between the base and the middle, suggesting roles in adventitious root formation in *C. japonica*. Although alterations in expression levels of *SCR* homologs followed similar patterns in the base and the middle, these were greater at the base.

The genes *NCED* encodes an enzyme that catalyzes the degradation of the *cis*-epoxy carotenoids *cis*-neoxanthin and *cis*-violaxanthin to xanthoxin, which is a direct precursor for abscisic acid (ABA) synthesis, and therefore regulate ABA concentration [67, 68]. *NCED* and thus ABA have reported inhibitory roles in adventitious root formation [12]; a tomato mutant with a null mutation in the gene *LeNCED1* formed more adventitious roots than its wild-type counterparts [69], and change in endogenous ABA contents after excision differed markedly between easy- and difficult-to-root clones [70]. Similarly, we observed restored expression of *NCED* homologs at the base after marked decreases following excision. In addition, Abu-Abied et al. [71] showed that the ability to form adventitious roots decreases during the transition from juvenile to mature phase and *NIA* expression and related nitric oxide (NO) synthesis upon excision was greater in juvenile cuttings than in mature cuttings in *Eucalyptus grandis*. In the present study, the *NIA* homolog was markedly induced at 3 hpi in the base and the middle, but was maintained at a high level at the base only and returned to original levels in the middle. These data support important roles of *NIA* and thus NO in adventitious root formation.

## Conclusions

**Fig. 9:**
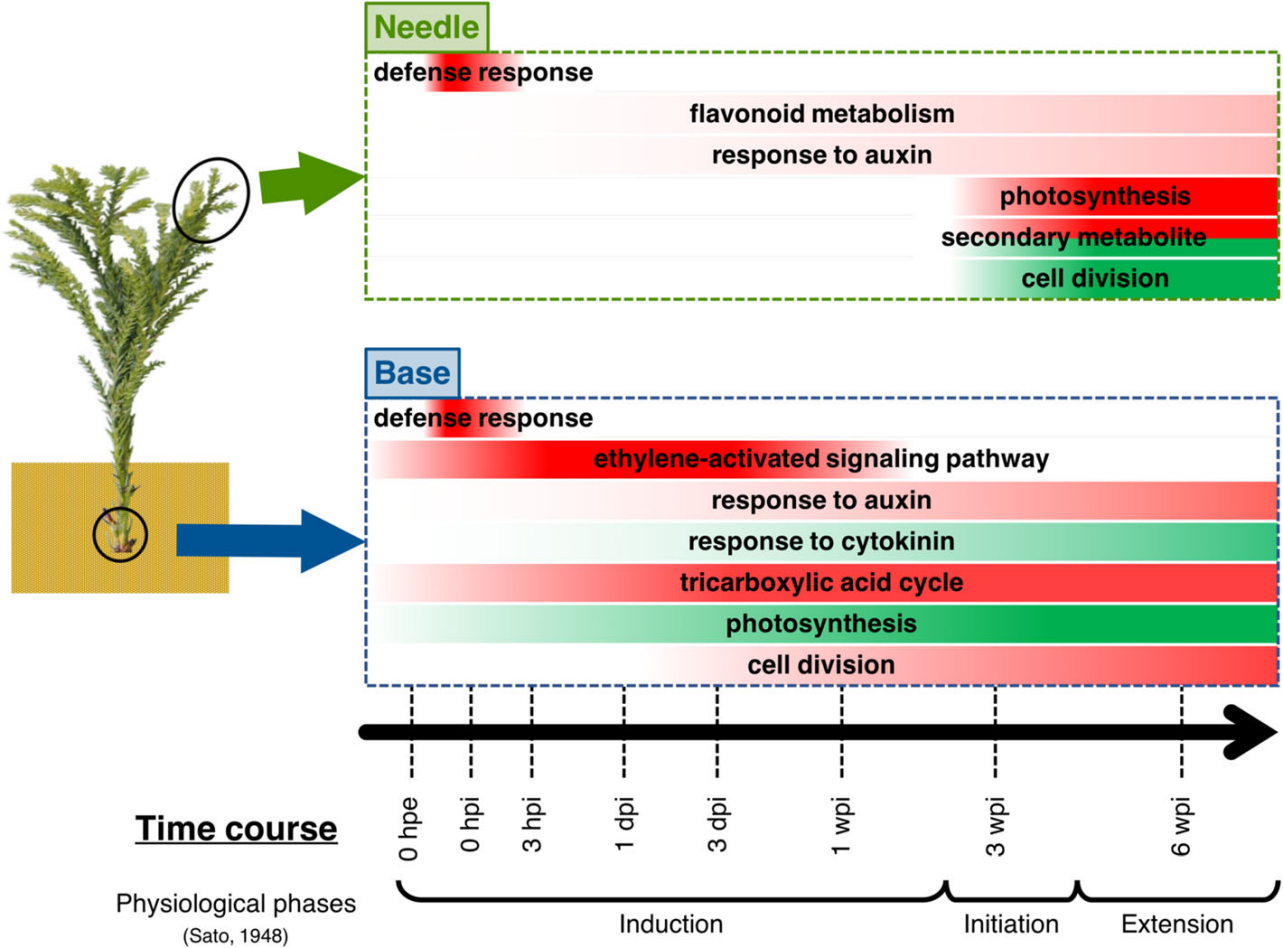
Major trends of gene expression during adventitious root formation in *C. japonica*. Color strengths of red and green indicates up- and down-regulation of gene expression level, respectively

In this study, we investigated transcriptome dynamics during adventitious root formation in cuttings of the coniferous species, *C. japonica* (Fig. 9). We then characterized temporal changes in gene expression in whole cuttings and confirmed the expression profiles of genes with reported roles in biological changes in adventitious root formation in various other plant species. Our results indicated transcriptional turning points for adventitious root formation at the base of cuttings. Expression behavior at the base of cuttings of energy and plant hormonal metabolism related genes generally supported previous biochemical and molecular biological observations of herbaceous plants, and comparisons of the expression behavior between the base and the middle of cuttings highlighted the importance of these genes for adventitious root formation. In needles, global gene expression changed in three phases. The expression of genes related to auxin metabolism and photosynthesis in needles indicated that they contributed to adventitious root formation at the base of cuttings. Although further biochemical and physiological research is required, our results offered a transcriptome insight into the function of needles for adventitious root formation in *C. japonica*. The present study represents a useful molecular biological basis for understanding the physiological mechanism of adventitious root formation in coniferous species.

## Additional files

Additional file 1: **Figure S1.** Validation of microarray data by qRT-PCR at the base. Bars represent the means ± standard errors of the means (SE) for three biological replicates. (DOCX 142 kb)

Additional file 2: **Figure S2.** Validation of microarray data by qRT-PCR in the middle. Bars represent the means ± standard errors of the means (SE) for three biological replicates. (DOCX 143 kb)

Additional file 3: **Figure S3.** Validation of microarray data by qRT-PCR in needles. Bars represent the means ± standard errors of the means (SE) for three biological replicates. (DOCX 141 kb)

Additional file 4: **Table S1.** Primers for qRT-PCR analyses. (XLSX 10 kb)

Additional file 5: **Table S2.** Annotation of genes in clusters in (A) “all”, (B) “base and middle” and (C) “needles”. (XLSX 1006 kb)

Additional file 6: **Table S3.** Functional annotation in clusters in (A) “all”, (B) “base and middle” and (C) “needles”. (XLSX 22 kb)

Additional file 7: **Table S4.** Annotation of the top 100 genes with the largest principal component 1 (PC1) coefficients. (XLSX 16 kb)

Additional file 8: **Table S5.** Functional enrichment annotations for the top 100 genes with the largest PC1. (XLSX 10 kb)

Additional file 9: **Table S6.** Annotation for adventitious root formation related genes included in list 2′ for “base and middle”. Gene names are given by DAVID. (XLSX 11 kb)

## Abbreviations

ABA: Abscisic acid
ACO: 1-Aminocyclopropane-1-carboxylate oxidase
ARFs: Auxin response factors
Cy3: Cyanine-3
DAVID: The Database for Annotation, Visualization and Integrated Discovery
dpi: Days post-insertion
ETO: Ethylene overproducer
GH3: Gretchen hagen 3
GO: Gene ontology
hpe: Hours post-excision
hpi: Hours post-insertion
NCED: Nine-cis-epoxycarotenoid dioxygenase
NIA: Nitrate reductase
NO: Nitric oxide
PCA: Principal component analysis
PIN: Pin formed
SAUR: Aux/iaa, small auxin up rna
SCR: Scarecrow
SHR: Short-root
TYPE-B RR: Cytokinin type-b response regulator; wpi: Weeks post-insertion

## References

[1] Bellini C, Pacurar DI, Perrone I (2014). Adventitious roots and lateral roots: similarities and differences. Annu Rev Plant Biol.

[2] de Klerk GJ, van der Krieken W, de Jong JC (1999). Review the formation of adventitious roots: new concepts, new possibilities. In Vitro Cell Dev Biol Plant.

[3] Pop TI, Pamfil D, Bellini C (2011). Auxin control in the formation of adventitious roots. Not Bot Hort Agrobot Cluj.

[4] Gutierrez L, Mongelard G, Floková K, Păcurar DI, Novák O, Staswick P, Kowalczyk M, Păcurar M, Demailly H, Geiss G, Bellini C (2012). Auxin controls Arabidopsis adventitious root initiation by regulating jasmonic acid homeostasis. Plant Cell.

[5] Ahkami AH, Melzer M, Ghaffari MR, Pollmann S, Javid MG, Shahinnia F, Hajirezaei MR, Druege U (2013). Distribution of indole-3-acetic acid in *Petunia hybrida* shoot tip cuttings and relationship between auxin transport, carbohydrate metabolism and adventitious root formation. Planta.

[6] Pacurar DI, Perrone I, Bellini C (2014). Auxin is a central player in the hormone cross-talks that control adventitious rooting. Physiol Plantarum.

[7] Haissig BE (1989). Carbohydrate relations during propagation of cuttings from sexually mature *Pinus banksiana* trees. Tree Physiol.

[8] Li MS, Leung DWM (2000). Starch accumulation is associated with adventitious root formation in hypocotyl cuttings of *Pinus radiata*. J Plant Growth Regul.

[9] Ahkami AH, Lischewski S, Haensch KT, Porfirova S, Hofmann J, Rolletschek H, Melzer M, Franken P, Hause B, Druege U, Hajirezaei MR (2009). Molecular physiology of adventitious root formation in *Petunia hybrida* cuttings: involvement of wound response and primary metabolism. New Phytol.

[10] Klopotek Y, Haensch KT, Hause B, Hajirezaei MR, Druege U (2010). Dark exposure of petunia cuttings strongly improves adventitious root formation and enhances carbohydrate availability during rooting in the light. J Plant Physiol.

[11] Li SW, Xue LG, Xu SJ, Feng HY, An LZ (2009). Mediators, genes and signaling in adventitious rooting. Bot Rev.

[12] da Costa CT, de Almeida MR, Ruedell CM, Schwambach J, Maraschin FS, Fett-Neto AG (2013). When stress and development go hand in hand: main hormonal controls of adventitious rooting in cuttings. Front Plant Sci.

[13] Steffens B, Rasmussen A (2016). The physiology of adventitious roots. Plant Physiol.

[14] Druege U, Franken P, Hajirezaei MR (2016). Plant hormone homeostasis, signaling, and function during adventitious root formation in cuttings. Front Plant Sci.

[15] Sorin C, Bussell JD, Camus I, Ljung K, Kowalczyk M, Geiss G, McKhann H, Garcion C, Vaucheret H, Sandberg G, Bellini C (2005). Auxin and light control of adventitious rooting in Arabidopsis require ARGONAUTE1. Plant Cell.

[16] Inukai Y, Sakamoto T, Ueguchi-Tanaka M, Shibata Y, Gomi K, Umemura I, Hasegawa Y, Ashikari M, Kitano H, Matsuoka M (2005). *Crown rootless1*, which is essential for crown root formation in rice, is a target of an AUXIN RESPONSE FACTOR in auxin signaling. Plant Cell.

[17] Klopotek Y, Franken P, Klaering HP, Fischer K, Hause B, Hajirezaei MR, Druege U (2016). A higher sink competitiveness of the rooting zone and invertases are involved in dark stimulation of adventitious root formation in *Petunia hybrida* cuttings. Plant Sci.

[18] Alba R, Fei Z, Payton P, Liu Y, Moore SL, Debbie P, Cohn J, D'Ascenzo M, Gordon JS, Rose JKC, Martin G, Tanksley SD, Bouzayen M, Jahn MM, Giovannoni J (2004). ESTs, cDNA microarrays, and gene expression profiling: tools for dissecting plant physiology and development. Plant J.

[19] Quan JE, Meng S, Guo EH, Zhang S, Zhao Z, Yang XT (2017). *De novo* sequencing and comparative transcriptome analysis of adventitious root development induced by exogenous indole-3-butyric acid in cuttings of tetraploid black locust. BMC Genomics.

[20] Ahkami A, Scholz U, Steuernagel B, Strickert M, Haensch KT, Druege U, Reinhardt D, Nouri E, von Wirén N, Franken P, Hajirezaei MR (2014). Comprehensive transcriptome analysis unravels the existence of crucial genes regulating primary metabolism during adventitious root formation in *Petunia hybrida*. PLoS One.

[21] Jaramillo-Correa JP, Verdu M, González-Martínez SC (2010). The contribution of recombination to heterozygosity differs among plant evolutionary lineages and life-forms. BMC Evol Biol.

[22] Ritchie GA (1991). The commercial use of conifer rooted cuttings in forestry: a world overview. New For.

[23] Toda R (1974). Vegetative propagation in relation to Japanese forest tree improvement. New Zeal J For Sci.

[24] Miyajima H (1951). On the relation between the races of *Cryptomeria japonica* and their root-formation in the cuttings. Sci Bull Fac Agr Kyushu Univ.

[25] Satoo T, Negishi K, Nakamura K (1953). Relations between the rooting and the age of the tree from which cuttings are taken. An experiment using clonal materials. J Jpn For Soc.

[26] Jull LG, Warren SL, Blazich FA (1994). Rooting ‘Yoshino’ Cryptomeria stem cuttings as influenced by growth stage, branch order, and IBA treatment. Hortscience.

[27] Shibuya T, Taniguchi T, Tsukuda S, Shiozaki S, Itagaki K (2014). Adventitious root formation of Japanese cedar (*Cryptomeria japonica* D. Don) cuttings is stimulated by soaking basal portion of cuttings in warmed water while cooling their apical portion. New Forest.

[28] Satoo S (1948). Origin of adventitious roots in Cryptomeria cuttings. Bull Tokyo Univ For.

[29] Brinker M, van Zyl L, Liu WB, Craig D, Sederoff RR, Clapham DH, von Arnold S (2004). Microarray analyses of gene expression during adventitious root development in *Pinus contorta*. Plant Physiol.

[30] Han H, Sun XM, Xie YH, Feng J, Zhang SG (2014). Transcriptome and proteome profiling of adventitious root development in hybrid larch (*Larix kaempferi* × *Larix olgensis*). BMC Plant Biol.

[31] Garrido G, Guerrero JR, Cano EA, Acosta M, Sánchez-Bravo J (2002). Origin and basipetal transport of the IAA responsible for rooting of carnation cuttings. Physiol Plant.

[32] Wilkerson EG, Gates RS, Zolnier S, Kester ST, Geneve RL (2005). Transpiration capacity in poinsettia cuttings at different rooting stages and the development of a cutting coefficient for scheduling mist. J Amer Soc Hort Sci.

[33] Gondo H, Ippei H, Toshimitsu K (1959). The relation between the leaf ratio and adventitious rooting rate in *Cryptomeria japonica*. Kyushu J For Res.

[34] Akashi T (1966). Rooting test in difficult-to-root clone of *Cryptomeria japonica*. Kyushu J For Res.

[35] Kuroki S (1967). Rooting test in *Cryptomeria japonica* (part 1) -the relation of callus formation and light intensity. Kyushu J For Res.

[36] Mishima K, Hirao T, Tsubomura M, Tamura M, Kurita M, Nose M, Hanaoka S, Takahashi M, Watanabe A (2018). Identification of novel putative causative genes and genetic marker for male sterility in Japanese cedar (*Cryptomeria japonica* D.Don). BMC Genomics.

[37] Huang DW, Sherman BT, Lempicki RA (2009). Systematic and integrative analysis of large gene lists using DAVID bioinformatics resources. Nat Protoc.

[38] Mishima K, Fujiwara T, Iki T, Kuroda K, Yamashita K, Tamura M, Fujisawa Y, Watanabe A (2014). Transcriptome sequencing and profiling of expressed genes in cambial zone and differentiating xylem of Japanese cedar (*Cryptomeria japonica*). BMC Genomics.

[39] Nose M, Watanabe A (2014). Clock genes and diurnal transcriptome dynamics in summer and winter in the gymnosperm Japanese cedar (*Cryptomeria japonica* (L.f.) D.Don). BMC Plant Biol.

[40] Kevers C, Hausman JF, Faivre-Rampant O, Evers D, Gaspar T (1997). Hormonal control of adventitious rooting: progress and questions. J Appl Bot.

[41] Rapaka VK, Bessler B, Schreiner M, Druege U (2005). Interplay between initial carbohydrate availability, current photosynthesis, and adventitious root formation in *Pelargonium* cuttings. Plant Sci.

[42] Tombesi S, Palliotti A, Poni S, Farinelli D (2015). Influence of light and shoot development stage on leaf photosynthesis and carbohydrate status during the adventitious root formation in cuttings of *Corylus avellana* L. Front Plant Sci.

[43] Machida H, Ooishi A, Hosoi T, Komatsu H, Kamota F (1977). Studies on photosynthesis in cuttings during propagation: I. Changes in the rate of apparent photosynthesis in the cuttings of several plants after planting. J Jpn Soc. Hort Sci.

[44] Smalley TJ, Dirr MA, Armitage AM, Wood BW, Teskey RO, Severson RF (1991). Photosynthesis and leaf water, carbohydrate, and hormone status during rooting of stem cuttings of *Acer Rubrum*. J Amer Soc Hort Sci.

[45] Negishi K, Satoo T (1956). Photosynthesis, respiration and consumption of reserves of Sugi (*Cryptomeria japonica*) cuttings. J Jpn For Soc.

[46] Li SW, Shi RF, Leng Y (2015). *De Novo* characterization of the mung bean transcriptome and transcriptomic analysis of adventitious rooting in seedlings using RNA-Seq. PLoS One.

[47] Villacorta-Martín C, Sánchez-García AB, Villanova J, Cano A, van de Rhee M, de Haan J, Acosta M, Passarinho P, Pérez-Pérez JM (2015). Gene expression profiling during adventitious root formation in carnation stem cuttings. BMC Genomics.

[48] Plaxton WC, Podestá FE (2006). The functional organization and control of plant respiration. CRC Cr Rev Plant Sci.

[49] Seo M, Akaba S, Oritani T, Delarue M, Bellini C, Caboche M, Koshiba T (1998). Higher activity of an aldehyde oxidase in the auxin-overproducing *superroot1* mutant of *Arabidopsis thaliana*. Plant Physiol.

[50] Tiwari SB, Hagen G, Guilfoyle T (2003). The roles of auxin response factor domains in auxin-responsive transcription. Plant Cell.

[51] Li Y, Wu YH, Hagen G, Guilfoyle T (1999). Expression of the auxin-inducible GH3 promoter/GUS fusion gene as a useful molecular marker for auxin physiology. Plant Cell Physiol.

[52] Liu SP, Wang JR, Wang L, Wang XF, Xue YH, Wu P, Shou HX (2009). Adventitious root formation in rice requires OsGNOM1 and is mediated by the OsPINs family. Cell Res.

[53] Sukumar P, Maloney GS, Muday GK (2013). Localized induction of the ATP-binding cassette B19 auxin transporter enhances adventitious root formation in Arabidopsis. Plant Physiol.

[54] Müller B, Sheen J (2008). Cytokinin and auxin interplay in root stem-cell specification during early embryogenesis. Nature.

[55] Moubayidin L, Di Mambro R, Sabatini S (2009). Cytokinin-auxin crosstalk. Trends Plant Sci.

[56] Ramírez-Carvajal GA, Morse AM, Dervinis C, Davis JM (2009). The cytokinin type-B response regulator PtRR13 is a negative regulator of adventitious root development in *Populus*. Plant Physiol.

[57] Druege U, Franken P, Lischewski S, Ahkami AH, Zerche S, Hause B, Hajirezaei MR (2014). Transcriptomic analysis reveals ethylene as stimulator and auxin as regulator of adventitious root formation in petunia cuttings. Front Plant Sci.

[58] Veloccia A, Fattorini L, Della Rovere F, Sofo A, D'angeli S, Betti C, Falasca G, Altamura MM (2016). Ethylene and auxin interaction in the control of adventitious rooting in *Arabidopsis thaliana*. J Exp Bot.

[59] Cheong YH, Chang HS, Gupta R, Wang X, Zhu T, Luan S (2002). Transcriptional profiling reveals novel interactions between wounding, pathogen, abiotic stress, and hormonal responses in Arabidopsis. Plant Physiol.

[60] Wang KLC, Li H, Ecker JR (2002). Ethylene biosynthesis and signaling networks. Plant Cell.

[61] Negi S, Sukumar P, Liu X, Cohen JD, Muday GK (2010). Genetic dissection of the role of ethylene in regulating auxin-dependent lateral and adventitious root formation in tomato. Plant J.

[62] Wang KLC, Yoshida H, Lurin C, Ecker JR (2004). Regulation of ethylene gas biosynthesis by the *Arabidopsis* ETO1 protein. Nature.

[63] Yoshida H, Nagata M, Saito K, Kevin WLC, Ecker JR (2005). *Arabidopsis* ETO1 specifically interacts with and negatively regulates type 2 1-aminocyclopropane-1-carboxylate synthases. BMC Plant Biol.

[64] Christians MJ, Gingerich DJ, Hansen M, Binder BM, Kieber JJ, Vierstra RD (2009). The BTB ubiquitin ligases ETO1, EOL1 and EOL2 act collectively to regulate ethylene biosynthesis in Arabidopsis by controlling type-2 ACC synthase levels. Plant J.

[65] Guan L, Murphy AS, Peer WA, Gan LJ, Li Y, Cheng ZM (2015). Physiological and molecular regulation of adventitious root formation. CRC Cr Rev Plant Sci.

[66] Helariutta Y, Fukaki H, Wysocka-Diller J, Nakajima K, Jung J, Sena G, Hauser MT, Benfey PN (2000). The *SHORT-ROOT* gene controls radial patterning of the *Arabidopsis* root through radial signaling. Cell.

[67] Iuchi S, Kobayashi M, Taji T, Naramoto M, Seki M, Kato T, Tabata S, Kakubari Y, Yamaguchi-Shinozaki K, Shinozaki K (2001). Regulation of drought tolerance by gene manipulation of 9-*cis*-epoxycarotenoid dioxygenase, a key enzyme in abscisic acid biosynthesis in *Arabidopsis*. Plant J.

[68] Xiong LM, Zhu JK (2003). Regulation of abscisic acid biosynthesis. Plant Physiol.

[69] Thompson AJ, Thorne ET, Burbidge A, Jackson AC, Sharp RE, Taylor IB (2004). Complementation of *notabilis*, an abscisic acid-deficient mutant of tomato: importance of sequence context and utility of partial complementation. Plant Cell Environ.

[70] Noiton D, Vine JH, Mullins MG (1992). Endogenous indole-3-acetic acid and abscisic acid in apple microcuttings in relation to adventitious root formation. Plant Growth Regul.

[71] Abu-Abied M, Szwerdszarf D, Mordehaev I, Levy A, Rogovoy Stelmakh O, Belausov E, Yaniv Y, Uliel S, Katzenellenbogen M, Riov J, Ophir R, Sadot E (2012). Microarray analysis revealed upregulation of nitrate reductase in juvenile cuttings of Eucalyptus grandis, which correlated with increased nitric oxide production and adventitious root formation. Plant J.

