# Supplementary material for: Transcriptome dynamics of rooting zone and aboveground parts of cuttings during adventitious root formation in Cryptomeria japonica D. Don
Source: BMC Plant Biol. 2018 Sep 19;18:201. doi: 10.1186/s12870-018-1401-7 (PMC6148763; doi:10.1186/s12870-018-1401-7)
Supplement: Supplementary file 1 — Figure S1. Validation of microarray data by qRT-PCR at the base. Bars represent the means ± standard errors of the means (SE) for three biological replicates. (DOCX 142 kb) [file 12870_2018_1401_MOESM1_ESM.docx]

**Additional file 1: Figure S1. Validation of microarray data by qRT-PCR in the base.**

Bars represent the means ± standard errors of the means (SE) for three biological replicates. (DOCX 142 KB)
