# Supplementary material for: Transcriptome dynamics of rooting zone and aboveground parts of cuttings during adventitious root formation in Cryptomeria japonica D. Don
Source: BMC Plant Biol. 2018 Sep 19;18:201. doi: 10.1186/s12870-018-1401-7 (PMC6148763; doi:10.1186/s12870-018-1401-7)
Supplement: Supplementary file 2 — Figure S2. Validation of microarray data by qRT-PCR in the middle. Bars represent the means ± standard errors of the means (SE) for three biological replicates. (DOCX 143 kb) [file 12870_2018_1401_MOESM2_ESM.docx]

**Additional file 2: Figure S2. Validation of microarray data by qRT-PCR in the middle.**

Bars represent the means ± standard errors of the means (SE) for three biological replicates. (DOCX 144 KB)
